# Characterization and therapeutic potential of DepZ57, a stable depolymerase targeting hypervirulent K57 *Klebsiella pneumoniae*

**DOI:** 10.1128/aem.02535-25

**Published:** 2026-06-10

**Authors:** Sixiang Xu, Mengmeng Su, Xiaoyue Li, Pei Li, Xiangkuan Zheng, Huanxin Fang, Yilin Fang, Peifan Liu, Long Chen, Zihao Pan, Zhongming Tan, Hong Du, Wei Zhang

**Affiliations:** 1 Sanya Institute of Nanjing Agricultural University, MOE Joint International Research Laboratory of Animal Health and Food Safety, College of Veterinary Medicine, Nanjing Agricultural University, Key Lab of Animal Bacteriology, Ministry of Agriculture, Nanjing, China; 2 College of Life Sciences and Food Engineering, Hebei University of Engineering 117798, Handan, China; 3 Department of Clinical Laboratory, Zhangjiagang Hospital Affiliated to Soochow University 598720 , Zhangjiagang, China; 4 NHC Key Laboratory of Enteric Pathogenic Microbiology, Jiangsu Provincial Center for Disease Control and Prevention 12666, Nanjing, China; 5 Department of Clinical Laboratory, the Second Affiliated Hospital of Soochow University 105860, Suzhou, China; Universidad de los Andes, Bogotá, Colombia

**Keywords:** *Klebsiella pneumoniae*, bacteriophage, depolymerase, K57 serotype, stability assessment, *In vivo* efficacy

## Abstract

*Klebsiella pneumoniae* (*K. pneumoniae*) is recognized as a significant opportunistic pathogen capable of infecting both humans and animals. The emergence of multidrug-resistant strains presents a severe challenge to current antimicrobial therapies, necessitating the development of alternative treatments such as bacteriophages and their encoded enzymes. In this study, a polysaccharide depolymerase, designated DepZ57, was identified, expressed, and characterized from the K57-specific lytic phage vB_Kp_Z57. Bioinformatic analysis indicated that DepZ57 is a hydrophilic protein with a theoretical isoelectric point of 6.27 and adopts a typical β-helix structure. The purified depolymerase exhibited high physicochemical stability across a broad pH range (2.0–11.0) and temperature range (4°C–70°C). *In vitro*, the K57 capsule was degraded by DepZ57 at a minimum effective concentration ranging from 0.04 to 0.4 μg/mL, which subsequently sensitized the bacteria to macrophage phagocytosis and complement-mediated serum killing. In a lethal systemic mouse infection model induced by intraperitoneal injection, a 100% survival rate was achieved following the administration of 50 μg of DepZ57, compared with a 60% survival rate observed with the phage treatment. Bacterial burdens were effectively reduced by both treatments. Notably, the bacterial loads in the blood, lungs, and liver were significantly decreased in the DepZ57 treatment group. Specifically, the bacterial load in the blood was completely eliminated, and the bacterial loads in the liver and lungs were reduced by more than 99%. Histopathological analysis confirmed that DepZ57 treatment effectively prevented hepatic necrosis and pulmonary inflammatory infiltration. Collectively, these findings demonstrate the *in vivo* efficacy and stability of DepZ57, suggesting it may represent a viable candidate for the control of *K. pneumoniae* infections.

**IMPORTANCE:**

The emergence of multidrug-resistant and hypervirulent *Klebsiella pneumoniae* represents a severe threat to human health and the dairy industry. Capsular polysaccharide (CPS) is the primary virulence factor that shields *K. pneumoniae* from host immune clearance, and the hypervirulent K57 serotype is frequently linked to severe invasive infections. Phage-derived depolymerases have emerged as promising antivirulence agents capable of specifically dismantling bacterial capsules without inducing bacterial resistance. Here, we characterized a novel, highly stable phage depolymerase, DepZ57, which exhibits robust tolerance to extreme pH and temperature conditions and specifically targets K57-type CPS. Distinct from the parental phage, DepZ57 provides full protection against lethal K57 *K. pneumoniae* infection *in vivo* and effectively alleviates infection-induced tissue damage. This work highlights the potential of phage depolymerases as stable, safe, and efficient nonantibiotic therapeutics for the prevention and control of hypervirulent *K. pneumoniae* infections.

## INTRODUCTION

*Klebsiella pneumoniae (K. pneumoniae*) is a clinically important gram-negative opportunistic pathogen with a broad host range, causing severe infections in both humans and animals (1). In the dairy industry, it is a primary cause of bovine mastitis, leading to significant economic losses due to reduced milk yield (2). The clinical and veterinary significance of this pathogen has been further amplified by its high prevalence and the rapid emergence of multidrug-resistant (MDR) strains driven by extensive antibiotic use (3). Of particular concern is the increasing resistance to last-resort antibiotics, such as carbapenems and polymyxins, which critically compromise conventional treatment options (4, 5). At the same time, hypervirulent *K. pneumoniae* (hvKP) has garnered significant attention owing to its capacity to cause severe infections even in immunocompetent individuals. Among the hypervirulence-associated capsular types, the K57 serotype has been identified as one of the serotypes strongly linked to hvKP (6). Historically, hypermucoviscosity was considered the defining characteristic of hvKP. However, with advancing research, the identification standard has evolved (7). Currently, hvKP is primarily determined by the presence of five specific biomarkers: *iucA*, *iroB*, *peg-344*, *rmpA*, and *rmpA2* (8). Nevertheless, the expression of capsular polysaccharide (CPS) remains a critical determinant of virulence. The roles of CPS are multifaceted. For example, it serves as a physical barrier that helps bacteria evade host immune phagocytosis. Additionally, CPS promotes biofilm formation, thereby protecting bacteria from antibiotics (9). Of particular concern is the increasing convergence of hypervirulence and multidrug resistance. Evidence indicates that carbapenem-resistant hvKP has spread globally, posing a severe threat to both human and animal health (10). Consequently, the development of effective alternative or synergistic therapeutic strategies is urgently required.

Bacteriophage therapy has emerged as a promising strategy to control *K. pneumoniae* infections (11). Beyond whole phages, phage-encoded proteins, specifically depolymerases, have demonstrated considerable potential as antimicrobial agents (12). Compared to direct phage application, depolymerases offer distinct advantages, including no risk of horizontal gene transfer, a reduced tendency to induce bacterial resistance, and more predictable pharmacokinetics (13). Depolymerases exert their antibacterial effect by degrading key surface polysaccharides, including CPS, exopolysaccharides, and lipopolysaccharides. Their significant therapeutic efficacy has been demonstrated in several *in vivo* studies (14–17). However, the clinical application of depolymerases is often limited by their high specificity; a single depolymerase typically targets only one capsular serotype. Therefore, it is essential to continually identify and characterize novel, active enzymes targeting clinically prevalent and highly pathogenic serotypes.

While depolymerases targeting prevalent serotypes like K1 and K2 have been extensively characterized and proven capable of rescuing hosts from lethal infections, research on depolymerases targeting the hypervirulent K57 serotype remains limited (16, 18). Volozhantsev et al. identified two depolymerases with β-galactosidase activity that specifically hydrolyze the CPS of K57 *K. pneumoniae* (19). Li et al. characterized another K57-specific depolymerase, confirming its protective efficacy and anti-biofilm activity in a mouse model (20). However, a direct, mechanistic comparison between a purified depolymerase and its parental whole phage has not been extensively explored. Understanding the dynamic differences in how these two related agents interact with the pathogen and the host immune system is of great significance for developing and optimizing non-antibiotic therapies.

In this study, DepZ57, a highly stable depolymerase targeting the K57 serotype of *K. pneumoniae*, was identified and characterized. Subsequently, a systematic, direct comparison of DepZ57 with its parental lytic phage was conducted *in vitro* and in a lethal mouse sepsis model. The results suggest a mechanism in which DepZ57, by degrading the bacterial capsule, sensitizes the bacteria to complement-mediated killing and macrophage opsonophagocytosis. This strategy yielded a 100% protection rate in a mouse sepsis model, significantly outperforming the 60% protection rate observed with whole-phage therapy. These findings indicate the efficacy of DepZ57 in treating systemic infections and support its potential as a highly effective and safe clinical candidate.

## MATERIALS AND METHODS

### Animals

We purchased 60 female BALB/c mice from Jiangsu Qinglongshan Biological Technology Co., Ltd. The mice were 5 weeks old and weighed between 18 and 22 g.

### Bacterial strains and culture conditions

All *K. pneumoniae* strains used in this study were isolated and preserved in our laboratory. Their detailed information is listed in Table S1 at https://doi.org/10.6084/m9.figshare.32202348. Bacterial strains were cultured in Luria-Bertani (LB) broth (Thermo Fisher Scientific, UK). Species identification and capsular serotype determination were preliminarily performed by polymerase chain reaction (PCR). The primers used in this study are listed in Table S2 at https://doi.org/10.6084/m9.figshare.32202348.

### Genomic and phenotypic characterization of KP591

To evaluate the virulence traits of the challenge strain KP591, both genomic and phenotypic analyses were conducted. Genomic DNA was extracted using the FastPure Bacteria DNA Isolation Mini Kit (Vazyme, Nanjing, China) according to the manufacturer’s instructions. Whole-genome sequencing was performed on the Illumina NovaSeq PE150 platform by Beijing Novogene (Beijing, China). *De novo* genome assembly was carried out using Unicycler v0.5.0 (21). Capsular serotype prediction was performed using Kaptive 3.0, and specific virulence genes were identified using ABRicate v1.0.1 (https://github.com/tseemann/abricate) with the Virulence Factor Database (VFDB) as the reference database (22, 23). In parallel, the hypermucoviscosity (HV) phenotype was assessed using the string test. KP591 was streaked onto sheep blood agar plates and incubated at 37°C for 18–24 h. A single colony was touched with a 10 μL pipette tip to stretch a mucoviscous string. A positive result was defined as the formation of a viscous string exceeding 5 mm in length (18).

### Phage isolation and purification

Phage vB_Kp_Z57 was isolated from sewage samples collected from a hospital in eastern China. To remove particulate debris, sewage samples were centrifuged at 8,000 × *g* for 10 min, and the resulting supernatant was filtered through a 0.22-μm membrane filter. For phage enrichment, the filtrate was mixed with the host strain KP591 and incubated at 37°C for 12 h. The enrichment culture was subsequently centrifuged at 6,000 × *g* for 10 min, and the phage-containing supernatant was collected and passed through a 0.22-μm membrane filter. Phage isolation was performed using the double-layer agar method. To ensure phage purity, individual plaques were picked and subjected to repeated rounds of plaque purification until homogeneous plaque morphology was observed (24).

### Phage genome sequencing and annotation

Phage genome sequencing was performed as previously described (25). Briefly, genomic DNA was extracted using the λ Phage Genomic DNA Extraction Kit (Zomanbio, Beijing, China) according to the manufacturer’s instructions. Whole-genome sequencing was performed on the Illumina NovaSeq 6000 system (San Diego, CA, USA). The genome was assembled *de novo* using SPAdes v3.11.1 (26). Genome annotation was performed using the RAST server (27). The circular genomic map was generated using Proksee (28).

### Bioinformatics prediction of phage depolymerase

Putative depolymerase-encoding genes were predicted using the DePolymerase Predictor and DepoScope software (29, 30). Candidate protein sequences were queried against the NCBI database using BLASTp (31). The physicochemical properties of DepZ57, including molecular weight, theoretical isoelectric point (pI), instability index, and grand average of hydropathicity (GRAVY), were calculated using the ExPASy ProtParam server (https://web.expasy.org/protparam/). Conserved domains were analyzed using HHpred and Phyre2.2 (32, 33). Additionally, structural modeling was performed using AlphaFold 3 (34). Phylogenetic analysis based on the amino acid sequences of the tail fiber protein (TFP) was conducted using MEGA X, including putative depolymerase and 19 homologous proteins retrieved from the NCBI database (35). Multiple sequence alignment of the predicted depolymerase with previously reported K57-specific depolymerases was performed using Clustal Omega (EMBL-EBI) (36).

### Cloning, expression, and purification of depolymerase

The putative depolymerase-encoding gene was amplified by PCR using gene-specific primers (forward: CAGCAAATGGGTCGCGGATCCATGCTGAACGACTTCAACCAGC; reverse: GTGGTGGTGGTGGTGCTCGAGTTAGTCCCCAAGGTAGACCAGTCG. The purified PCR product was digested with *Bam*HI and *Xho*I and ligated into the pET-28a expression vector. The resulting recombinant plasmid was transformed into *Escherichia coli* BL21 (DE3)-competent cells. Transformed cells were cultured in LB medium until the optical density at 600 nm (OD_600_) reached approximately 0.6. Recombinant depolymerase expression was induced by the addition of 0.5 mM isopropyl-β-D-thiogalactopyranoside (IPTG) followed by incubation at 16°C for 12 h. The bacteria were harvested by centrifugation at 8,000 × *g* for 10 min at 4°C. The bacterial pellet was resuspended in lysis buffer (2 mM Na_3_PO_4_·12H_2_O, 50 mM NaCl, and 30 mM imidazole, pH 7.4) and disrupted by sonication. The lysate was clarified by centrifugation at 10,000 × *g* for 15 min at 4°C, and the supernatant containing soluble His-tagged depolymerase was collected. The recombinant depolymerase was purified using a Ni-NTA affinity column and eluted with a buffer containing 500 mM imidazole. The depolymerase was subsequently exchanged into phosphate-buffered saline (PBS) and concentrated using a 30-kDa molecular-weight-cutoff centrifugal ultrafiltration device. Depolymerase concentration was determined using a bicinchoninic acid (BCA) protein assay kit (Thermo Scientific, USA). The purity of the depolymerase was analyzed by 10% sodium dodecyl sulfate-polyacrylamide gel electrophoresis (SDS-PAGE).

### Depolymerase activity assay

Depolymerase activity was evaluated using a spot assay as previously described (18). Serial dilutions of purified DepZ57 ranging from 400 to 0.04 μg/mL were spotted onto double-layer agar plates overlaid with the indicator strain KP591. PBS was used as the negative control, and phage vB_Kp_Z57 served as the positive control. Depolymerase activity was indicated by the formation of semi-translucent halos surrounding the spotted areas. To further evaluate capsule degradation, strain KP591 was cultured in LB broth to the logarithmic phase (OD_600_ = 0.6). The cells were centrifuged at 5,000 × *g* for 5 min and washed three times with PBS. The bacterial pellet was resuspended in either 1 mL of PBS or 1 mL of DepZ57 (10 μg/mL) and incubated at 37°C for 2 h. Finally, the cells were collected by centrifugation at 5000 × *g* for 5 min to observe any morphological differences in the pellets.

### Host range and depolymerase spectrum analysis

The host range of phage vB_Kp_Z57 and the activity spectrum of the recombinant depolymerase were evaluated using the *K. pneumoniae* strains listed in Table S1 at https://doi.org/10.6084/m9.figshare.32202348. Exponential phase bacterial cultures (200 μL) were mixed with 5 mL of molten 0.5% LB soft agar and overlaid onto 1.5% LB agar plates. After solidification, 5 μL of either purified phage suspension or recombinant depolymerase solution was spotted onto the agar surface. Plates were incubated at 37°C for 12 h, and lytic zones or semi-translucent halos were subsequently examined (14).

### Stability characterization of DepZ57

The pH stability of the depolymerase was assessed across a broad pH range (pH 1.0–11.0). The purified protein was incubated in SM buffer (50 mM Tris-HCl, 100 mM NaCl, 10 mM MgSO_4_, and 0.01% gelatin) adjusted to specific pH values. Incubation was carried out at 37°C for 1 h. Regarding thermal stability, the depolymerase was incubated at specific temperatures (4°C, 20°C, 30°C, 40°C, 50°C, 60°C, and 70°C) for 1 h. Residual activity was subsequently determined using the spot assay.

### Macrophage phagocytosis assay

The effect of the depolymerase on the anti-phagocytic ability of KP591 was evaluated using murine macrophage RAW 264.7 cells, as described previously (36). The cells were cultured in DMEM supplemented with 10% fetal bovine serum (FBS) and maintained at 37°C in a 5% CO_2_ incubator. Cells were seeded into 24-well plates (5 × 10^5^/well) and cultured until a confluent monolayer formed. *K. pneumoniae* strain KP591 was cultured in LB broth to the logarithmic phase (OD_600_ = 0.6). The bacterial culture was centrifuged at 12,000 × *g* for 10 min, washed three times with PBS, resuspended, and diluted to a final concentration of 1 × 10^7^ CFU/mL in PBS. The bacterial suspension was treated with 10, 20, or 50 μg/mL of DepZ57 or an equal volume of PBS and incubated at 37°C for 2 h. After treatment, bacteria were washed three times with PBS and resuspended in FBS-free DMEM. The RAW 264.7 monolayers were washed three times with PBS. The pretreated bacterial suspensions (500 μL/well) were added to the macrophages at a bacterium-to-cell ratio of 10:1. The plates were centrifuged at 800 × *g* for 10 min at room temperature and incubated at 37°C with 5% CO_2_ for 2 h. The cells were washed three times with PBS and incubated in DMEM containing 100 μg/mL gentamicin for 2 h to eradicate extracellular bacteria. After five additional washes with PBS, the cells were lysed with 1 mL of sterile water by repeated pipetting. The cell lysates were subjected to 10-fold serial dilutions, and a 10 μL aliquot of each dilution was spotted onto LB agar plates. The plates were incubated at 37°C for 12 h to enumerate the intracellular CFU. The assay was performed in triplicate.

### Serum sensitivity assay

The serum bactericidal assay was performed with reference to previous work. Serum was collected from 5-week-old female BALB/c SPF mice and filtered through a 0.22-μm membrane. *K. pneumoniae* strain KP591 was cultured overnight in LB broth at 37°C and 180 rpm. The culture was then subcultured at a 1:100 ratio into fresh LB broth and grown to the logarithmic phase (OD_600_ = 0.6) under the same conditions. The cells were centrifuged at 12,000 × *g* for 10 min, washed three times with PBS, and resuspended in PBS to a final concentration of 5 × 10^7^ CFU/mL. The bacterial suspension was divided into five groups and mixed with PBS, or 10, 20, or 50 μg/mL of DepZ57, or phage vB_Kp_Z57 (MOI = 0.1). The mixtures were incubated at 37°C for 2 h. To remove residual depolymerase and phage, the samples were centrifuged at 5,000 × *g* for 10 min and washed three times with PBS. The bacterial pellets were then resuspended in PBS to a final concentration of 5 × 10^6^ CFU/mL. Each group was mixed with 75% (vol/vol) fresh mouse serum, heat-inactivated mouse serum (56°C, 30 min), or PBS, and incubated at 37°C for 2 h. The mixtures were serially diluted 10-fold in PBS, and a 10 μL aliquot of each dilution was spotted onto LB agar plates. The plates were incubated at 37°C for 12 h to determine the CFU. The assay was performed in triplicate for each group.

### Evaluation of therapeutic efficacy in a mouse model

A total of 60 female BALB/c mice were randomly divided into nine groups. Mice had free access to food and water throughout the experiment. *K. pneumoniae* strain KP591 was cultured in LB broth to the logarithmic phase (OD_600_ = 0.6), then centrifuged at 12,000 × *g* for 10 min, and washed three times with PBS. The bacteria were resuspended in PBS to a final concentration of 1 × 10^8^ CFU/mL. To establish infection, each mouse received an intraperitoneal injection of 100 μL of the bacterial suspension. Treatments were administered 2 h after infection. Mice in the control group received 100 μL of PBS by intraperitoneal injection. For phage treatment, 100 μL of phage suspension (1 × 10^9^ PFU/mL) was administered intraperitoneally. For depolymerase treatment, 50 μg of the purified depolymerase was injected intraperitoneally.

Survival analysis was conducted using Groups 1 to 3, with each group comprising 10 mice (*n* = 10). Group 1 served as the infection control (infected with KP591 and treated with PBS). Group 2 represented the phage treatment group (infected with KP591 and treated with 100 μL of phage). Group 3 represented the depolymerase treatment group (infected with KP591 and treated with 50 μg of depolymerase). Mice were monitored for 7 d. Subsequently, all surviving mice were euthanized.

Determination of bacterial loads and histopathological analysis were conducted using Groups 4–9, with each group comprising 5 mice (*n* = 5). Group 4 served as the positive control group (infected with KP591 and treated with PBS). Group 5 represented the phage treatment group (infected with KP591 and treated with phage). Group 6 represented the depolymerase treatment group (infected with KP591 and treated with depolymerase). Groups 7–9 served as non-infected safety controls: Group 7 was injected with 100 μL of PBS only; Group 8 was injected with 100 μL of phage only; and Group 9 was injected with 50 μg of depolymerase only. Mice in these groups were euthanized 12 h after infection. Blood samples were collected from the orbital sinus. The lungs, liver, spleen, and kidneys were harvested and homogenized. The homogenates and blood samples were subjected to 10-fold serial dilutions in PBS. A 10 μL aliquot of each dilution was spotted onto LB agar for colony counting using the drop plate method. Each sample was plated in triplicate. For histopathological analysis, lung and liver tissues were collected and fixed in 4% paraformaldehyde for 48 h. Tissue processing (dehydration, paraffin embedding, sectioning) and H&E staining were performed by Nanjing Youmeng Biotechnology Co., Ltd. (Nanjing, Jiangsu, China). The histopathological evaluation included assessment of structural integrity, inflammatory cell infiltration, congestion, and edema.

### Statistical analysis

Statistical analyses were performed using GraphPad Prism 8. Survival curves were analyzed using the log-rank (Mantel-Cox) test. Bacterial load and macrophage phagocytosis data were analyzed by one-way ANOVA followed by Dunnett’s multiple comparisons test. The serum killing assays were analyzed by two-way ANOVA followed by Sidak’s multiple comparisons test. Data are presented as mean ± standard deviation (SD). A *P* value of < 0.05 was considered statistically significant (*, *P* < 0.05; **, *P* < 0.01; ***, *P* < 0.001).

## RESULTS

### Characterization of *K. pneumoniae* KP591

**Fig 1:**
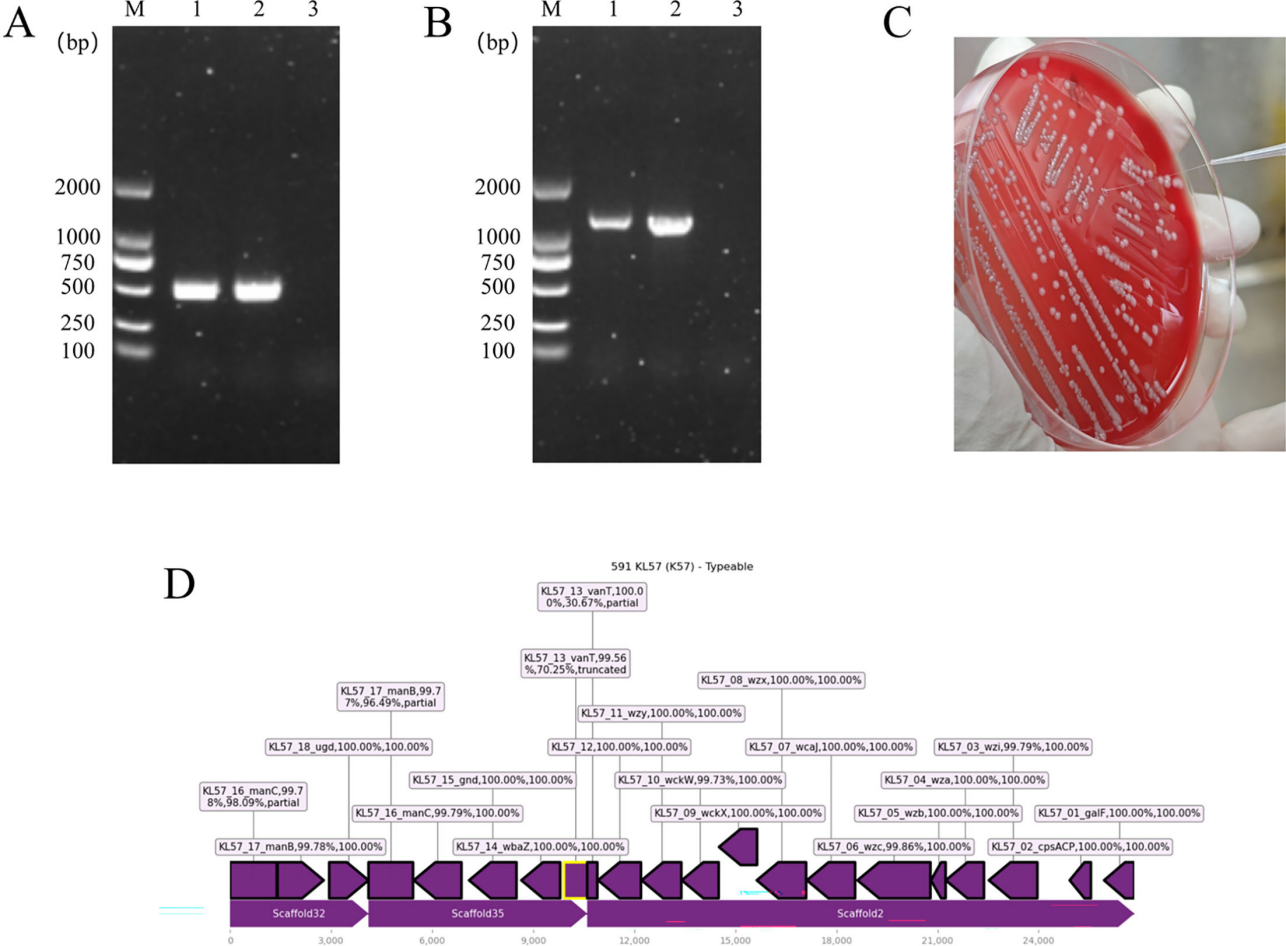
Characterization of *Klebsiella pneumoniae* strain KP591. (**A**) PCR identification of the *khe* gene in KP591. Lane M: DNA marker; Lane 1: KP591; Lane 2: Positive control; Lane 3: Negative control. (**B**) PCR identification of the K57 capsular serotype in KP591. Lane M: DNA marker; Lane 1: KP591; Lane 2: Positive control; Lane 3: Negative control. (**C**) Hypermucoviscosity phenotype assessed by the string test. (**D**) Capsular serotyping of KP591 performed by Kaptive 3.0. It displays the genetic map of the predicted K57 capsular polysaccharide locus and the identity percentage of individual genes compared to the reference database.

PCR amplification of the *khe* gene produced a distinct band at approximately 428 bp, consistent with the positive control, confirming the identification of KP591 as *K. pneumoniae* (Fig. 1A). Targeted PCR for the K57 serotype-specific gene yielded an expected amplicon of approximately 1037 bp, matching the positive control and establishing the serotype as K57 (Fig. 1B). KP591 was positive in the string test, forming a viscous string exceeding 5 mm in length. This hypermucoviscous phenotype is indicative of high virulence (Fig. 1C).

To validate the serotype and virulence profiles at the genomic level, whole-genome sequencing was performed on KP591. Kaptive 3.0 analysis confirmed the strain as serotype K57, with a match confidence of “Typeable” and a high sequence identity of 99.91% (Fig. 1D). Virulence gene screening identified four key biomarkers (*iucA*, *iroB*, *rmpA*, and *rmpA2*) within the KP591 genome. The simultaneous presence of these genetic markers classifies KP591 as a hvKP strain (see Table S3 at https://doi.org/10.6084/m9.figshare.32202348).

### Plaque morphology of vB_Kp_Z57

**Fig 2:**
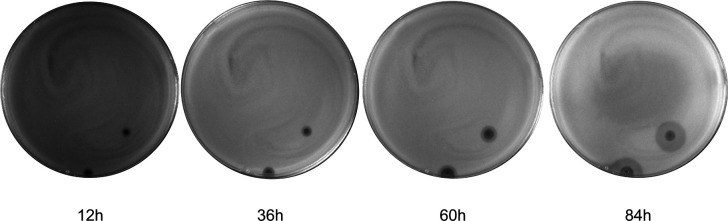
Temporal evolution of plaque morphology for phage vB_Kp_Z57 on host strain KP591. The formation of semi-translucent halos surrounding the clear lytic centers was observed, with the halo diameter exhibiting progressive expansion over time.

Using the double-layer agar method, phage vB_Kp_Z57 formed distinct, clear plaques on lawns of the host strain *K. pneumoniae* KP591 after incubation at 37°C. Notably, translucent halos surrounding the plaques were observed after 12 h of incubation, and these halos continued to expand over time (Fig. 2). This observation suggests that vB_Kp_Z57 encodes a depolymerase with capsular polysaccharide-degrading activity.

### Genome analysis of vB_Kp_Z57

**Fig 3:**
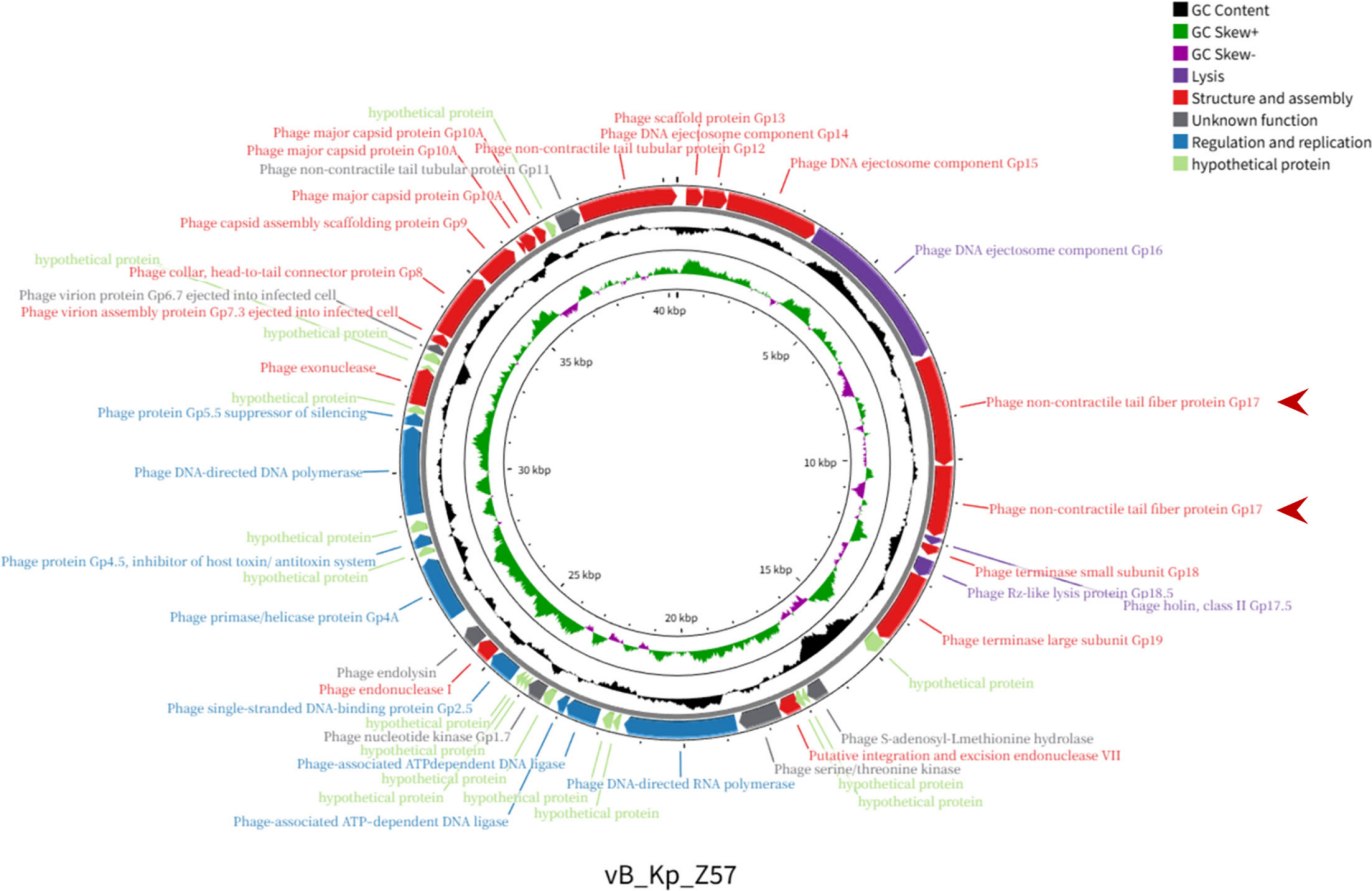
Circular genomic map of phage vB_Kp_Z57. The genome organization was visualized using Proksee. A total of 49 putative CDSs were mapped, comprising 34 annotated functional proteins and 15 hypothetical proteins. Red arrowheads indicate the positions of the putative tail fiber proteins.

The genome sequence of phage vB_Kp_Z57 (40,316 bp, 53.10% GC) was previously deposited in GenBank (accession number PP758980). In this study, we conducted a new analysis of the genome and found a previously unannotated gene. This gene encodes a putative non-contractile tail tubular protein (TTP). It begins at position 37,952 and crosses the ends of the sequence to finish at position 138. We combined the two parts of the gene to obtain the complete sequence for analysis, given that TTP often has both structural and lytic functions (37). The GenBank record is being updated to include this gene. With this addition, the genome contains 49 CDSs in total. These were classified into hypothetical, structural, and functional proteins based on RAST analysis (Fig. 3). Details for all CDSs are provided in Table S4 at https://doi.org/10.6084/m9.figshare.32202348.

### Analysis of predicted genes encoding depolymerases

**Fig 4:**
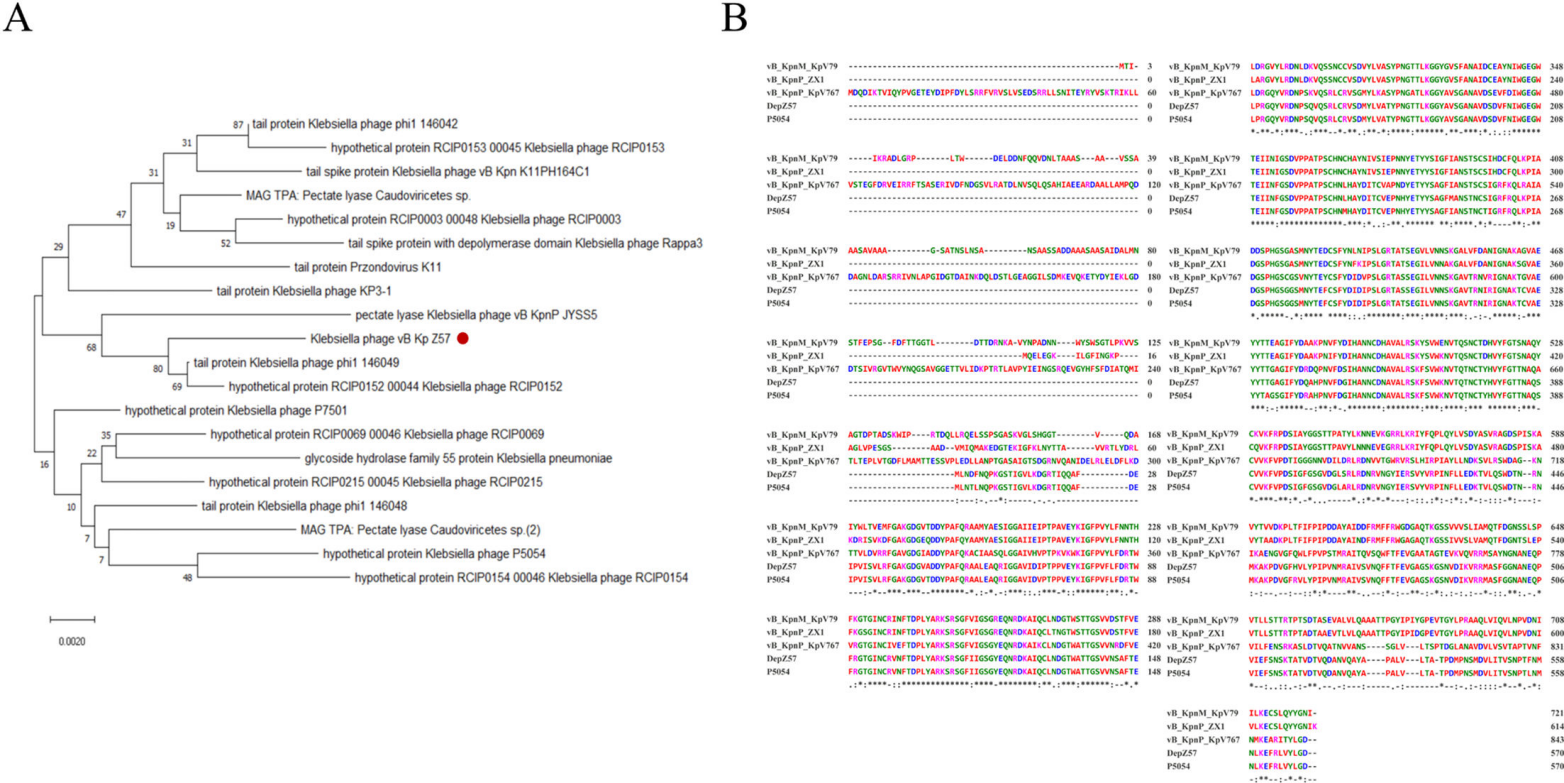
Bioinformatic characterization of the putative depolymerase DepZ57. (**A**) Phylogenetic analysis of DepZ57 was constructed using the Neighbor-Joining method in MEGA X (1,000 bootstrap replicates). The position of vB_Kp_Z57 is highlighted by a red dot. (**B**) Multiple sequence alignment of DepZ57 with previously reported K57-specific depolymerases.

**Fig 5:**
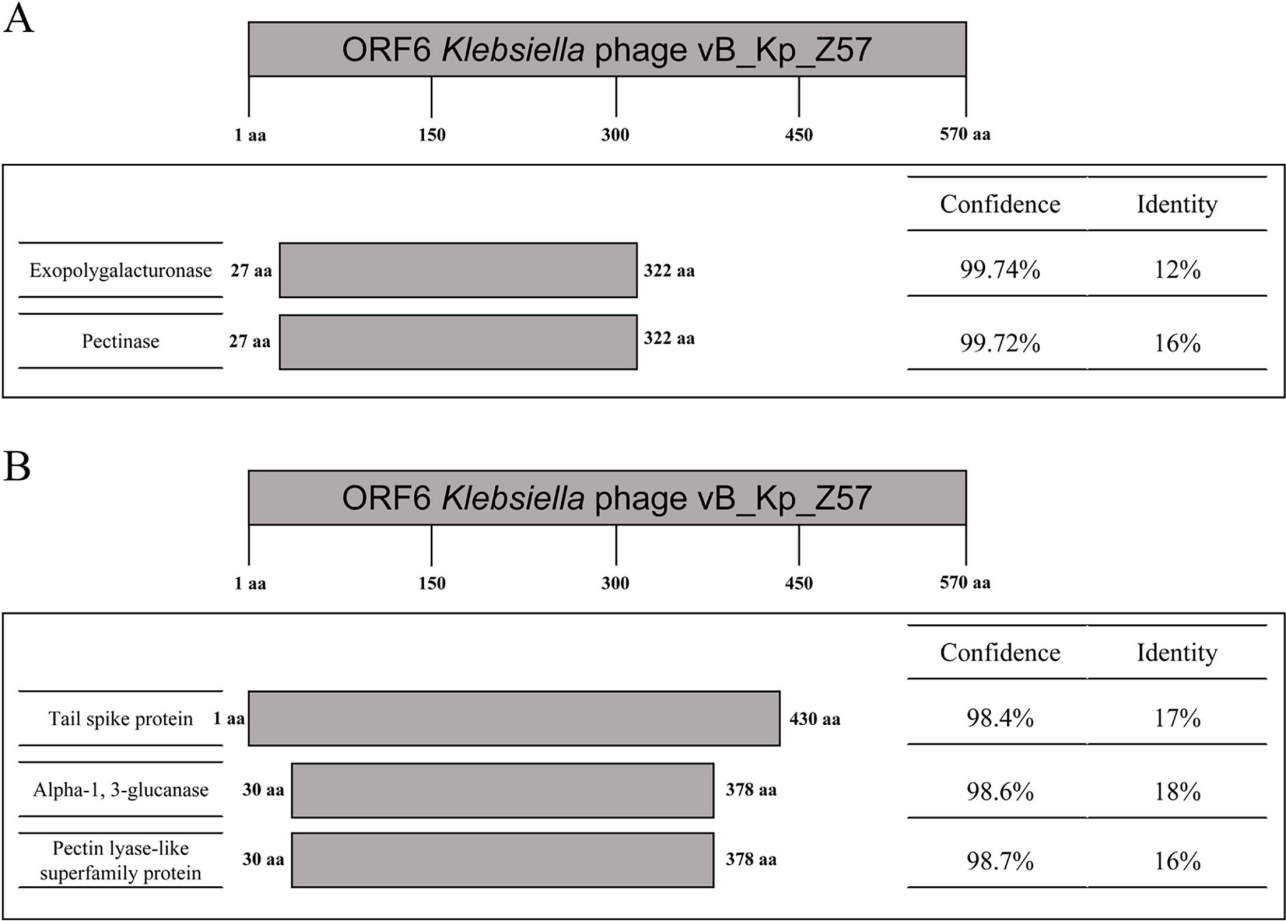
Functional annotation results of depolymerase DepZ57. (**A**) Prediction of conserved functional domains using HHpred. (**B**) Structural homology and domain analysis were performed using Phyre2.2.

Initial bioinformatic screening of the 49 CDSs in the vB_Kp_Z57 genome identified CDS5, CDS6, and CDS49 as putative depolymerase candidates (see Table S4 at https://doi.org/10.6084/m9.figshare.32202348). Given that depolymerases are typically tail fiber or tail spike proteins, RAST annotation classified both CDS5 and CDS6 as tail fiber proteins. Subsequent investigation focused on CDS6, as BLASTp analysis revealed high similarity (100% coverage; >97% identity) to 20 proteins, prominently including *Klebsiella* phage tail proteins and glycoside hydrolases (Fig. 4A). ProtParam predicted a molecular weight of approximately 62.5 kDa and a theoretical isoelectric point (pI) of 6.27. The calculated grand average of hydropathicity (GRAVY) of −0.223 and an instability index of 32.33 classify the protein as stable and hydrophilic. Moreover, CDS6 shares 97.54% identity (100% query coverage) with K57-Dpo8 (Accession No. XMN67129.1), a characterized depolymerase that targets *K. pneumoniae* serotype K57 (Fig. 4B). Structural homology searches using HHpred indicated that the region spanning amino acids 27–322 shares homology with polysaccharide hydrolases (Fig. 5A), while Phyre2.2 analysis consistently identified conserved domains typical of tail spike proteins and lyases (Fig. 5B). Protein modeling using AlphaFold3 revealed a typical β-helix structure with an ipTM score of 0.91, indicating high model confidence (see Fig. S1 at https://doi.org/10.6084/m9.figshare.32202348). Collectively, these results indicate that CDS6 encodes a polysaccharide depolymerase, designated DepZ57.

### Expression and activity of DepZ57

**Fig 6:**
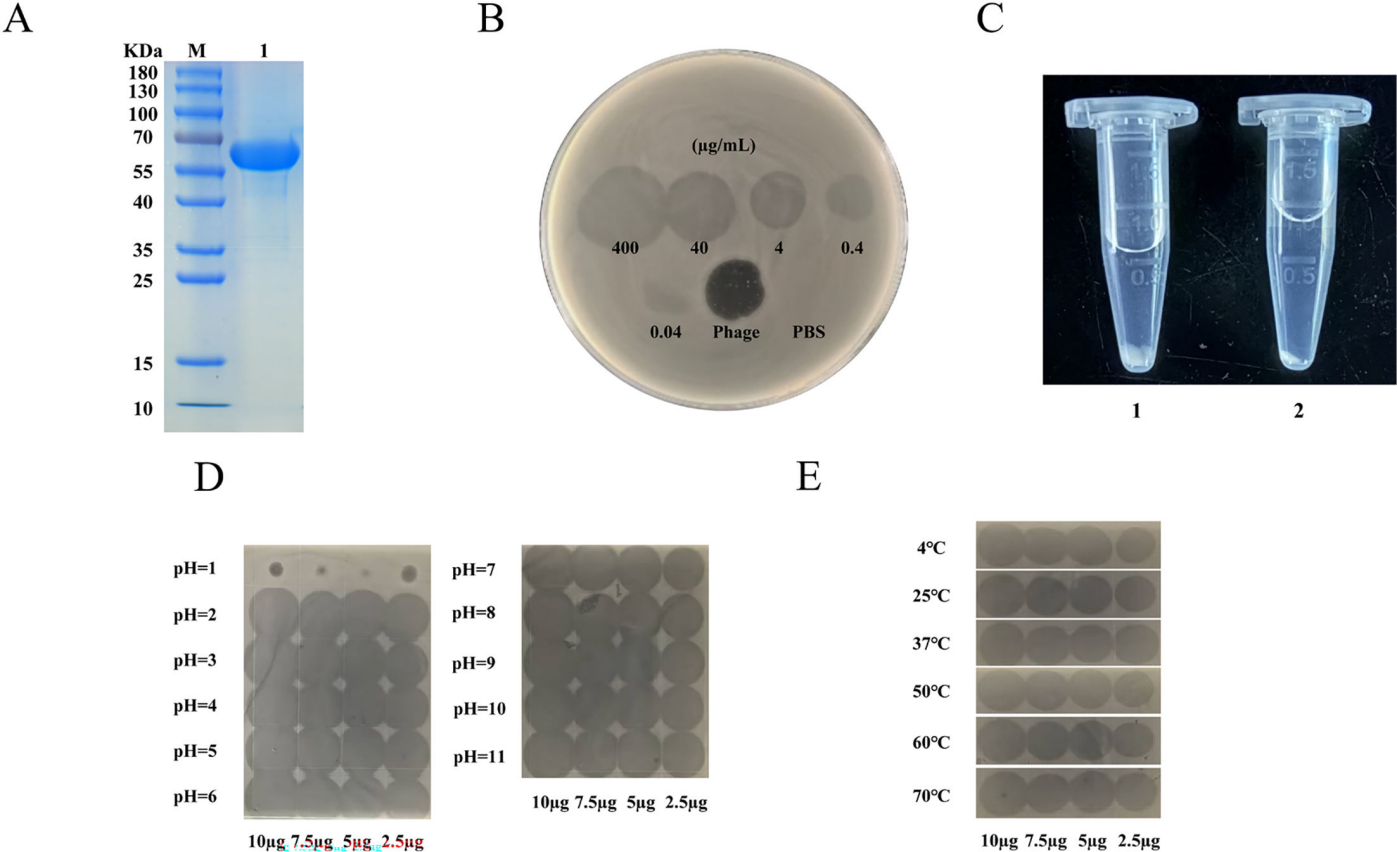
Purification and enzymatic characterization of the recombinant depolymerase DepZ57. (**A**) SDS-PAGE analysis of purified DepZ57. Lane M: protein marker (kDa); Lane 1: purified recombinant DepZ57. (**B**) Depolymerase activity assessed by spot assay on a lawn of *K. pneumoniae* KP591. Serial dilutions of DepZ57 ranging from 400 to 0.04 μg/mL were applied (5 μL/spot). Phage vB_Kp_Z57 and PBS served as the positive and negative controls, respectively. (**C**) Bacterial pellets of untreated and DepZ57-treated KP591. 1: untreated KP591; 2: KP591 treated with DepZ57 (10 μg/mL) for 2 h. (**D**) pH stability of DepZ57 was assessed from pH 1 to 11. (**E**) Thermal stability of DepZ57 was assessed following incubation at temperatures ranging from 4°C to 70°C.

The recombinant depolymerase DepZ57 was successfully expressed and purified, yielding a single prominent band of approximately 62 kDa on 10% SDS-PAGE (Fig. 6A). To evaluate its specific enzymatic activity, serially diluted DepZ57 was spotted onto *K. pneumoniae* KP591 lawns. The formation of distinct translucent halos indicated degradation of capsular polysaccharides. Halo size decreased in a dose-dependent manner, and the minimum effective concentration required to produce a visible halo was determined to be between 0.04 and 0.4 μg/mL (Fig. 6B). In the centrifugation assay, the untreated control group formed a loose bacterial pellet, whereas the DepZ57-treated group formed a tightly compact pellet (Fig. 6C). Assessment of pH stability showed that DepZ57 remained active across a broad range, from pH 2 to 11 (Fig. 6D). Thermal stability analysis revealed that at pH 7, DepZ57 maintained stable enzymatic activity following exposure to temperatures ranging from 4°C to 70°C (Fig. 6E). Spectrum analysis involving a diverse panel of 80 *K. pneumoniae* strains demonstrated that DepZ57 formed translucent degradation halos exclusively on K57 serotype strains (see Table S1 at https://doi.org/10.6084/m9.figshare.32202348).

### DepZ57 sensitizes KP591 to macrophage phagocytosis

**Fig 7:**
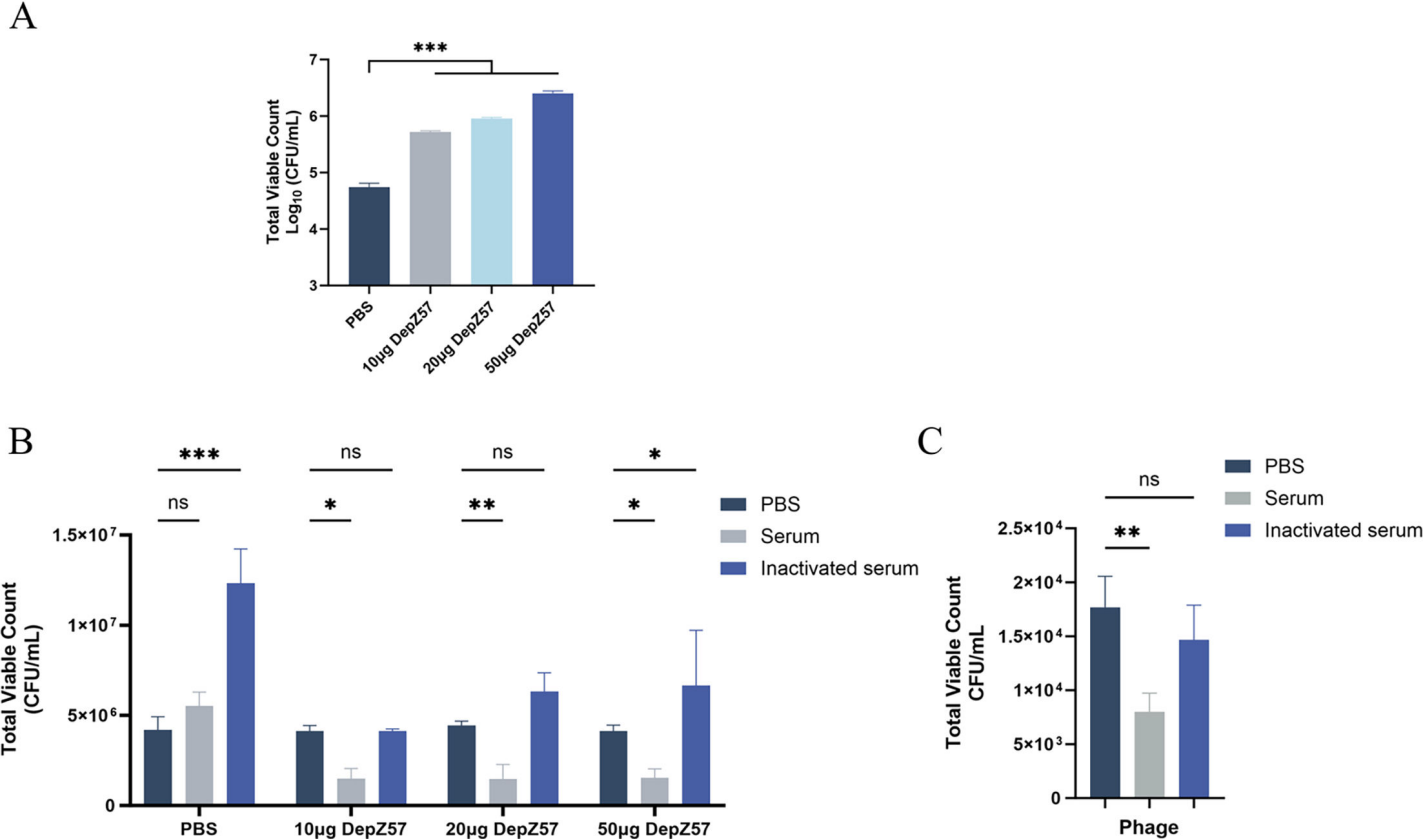
DepZ57 sensitizes *K. pneumoniae* KP591 to macrophage phagocytosis and serum killing. (**A**) DepZ57 enhances macrophage phagocytosis. RAW 264.7 macrophages phagocytosed an increased number of KP591 bacteria following treatment with DepZ57 at concentrations of 10, 20, and 50 μg/mL, and this effect was dose-dependent. (**B**) DepZ57 sensitizes KP591 to serum killing. Bacterial survival was assessed in PBS, active mouse serum, and heat-inactivated serum after DepZ57 pre-treatment. At all enzyme concentrations tested, bacterial loads were significantly reduced in the active serum group. (**C**) Phage vB_Kp_Z57 sensitizes KP591 to serum killing. Although phage-mediated sensitization to serum was observed, the majority of the reduction in bacterial counts was attributable to the direct lytic activity of the phage. Statistical significance was analyzed using two-way analysis of variance (ANOVA) followed by Sidak’s multiple comparisons test (*, *P* < 0.05, **, *P* < 0.01, ***, *P* < 0.001).

Treatment with DepZ57 increased the susceptibility of the K57 serotype *K. pneumoniae* strain KP591 to phagocytosis by murine RAW 264.7 macrophages (Fig. 7A). This effect positively correlated with enzyme concentration. In the PBS-treated control group, macrophages captured only 5.5 × 10^4^ CFU, reflecting a low baseline phagocytosis efficiency of 1.1%. Following enzymatic treatment, the number of internalized bacteria increased progressively. The 10 μg/mL and 20 μg/mL DepZ57 groups showed 5.2 × 10^5^ CFU (10.5% efficiency) and 8.9 × 10^5^ CFU (17.9% efficiency), respectively. Notably, the 50 μg/mL treatment group reached 2.5 × 10^6^ CFU, corresponding to a capture efficiency of 50.3%.

### vB_Kp_Z57 and DepZ57 sensitize KP591 to serum killing

Both phage vB_Kp_Z57 and DepZ57 enhanced the complement-mediated killing of *K. pneumoniae* KP591 in mouse serum. PBS-treated control bacteria proliferated in both active and heat-inactivated serum, confirming the inherent serum resistance of KP591. Pre-treatment with DepZ57 sensitized the bacteria to serum killing, resulting in a reduction of 62.9%–64.5% compared to the PBS-treated group. This effect was not dose-dependent. Similarly, phage pre-treatment induced serum sensitivity, with bacterial loads decreasing from 1.8 × 10^4^ CFU in the PBS-incubated group to 8 × 10^3^ CFU in the serum-incubated group (54.7% reduction) (Fig. 7B). Prior to serum exposure, however, phage pre-treatment alone had already reduced the bacterial load from 5 × 10^6^ CFU to 1.8 × 10^4^ CFU in the PBS control, representing a 99.6% reduction (Fig. 7C). This indicates that while the phage sensitizes bacteria to serum killing, primary bacterial elimination is driven by its direct lytic activity.

### Evaluation of vB_Kp_Z57 and DepZ57 protection

**Fig 8:**
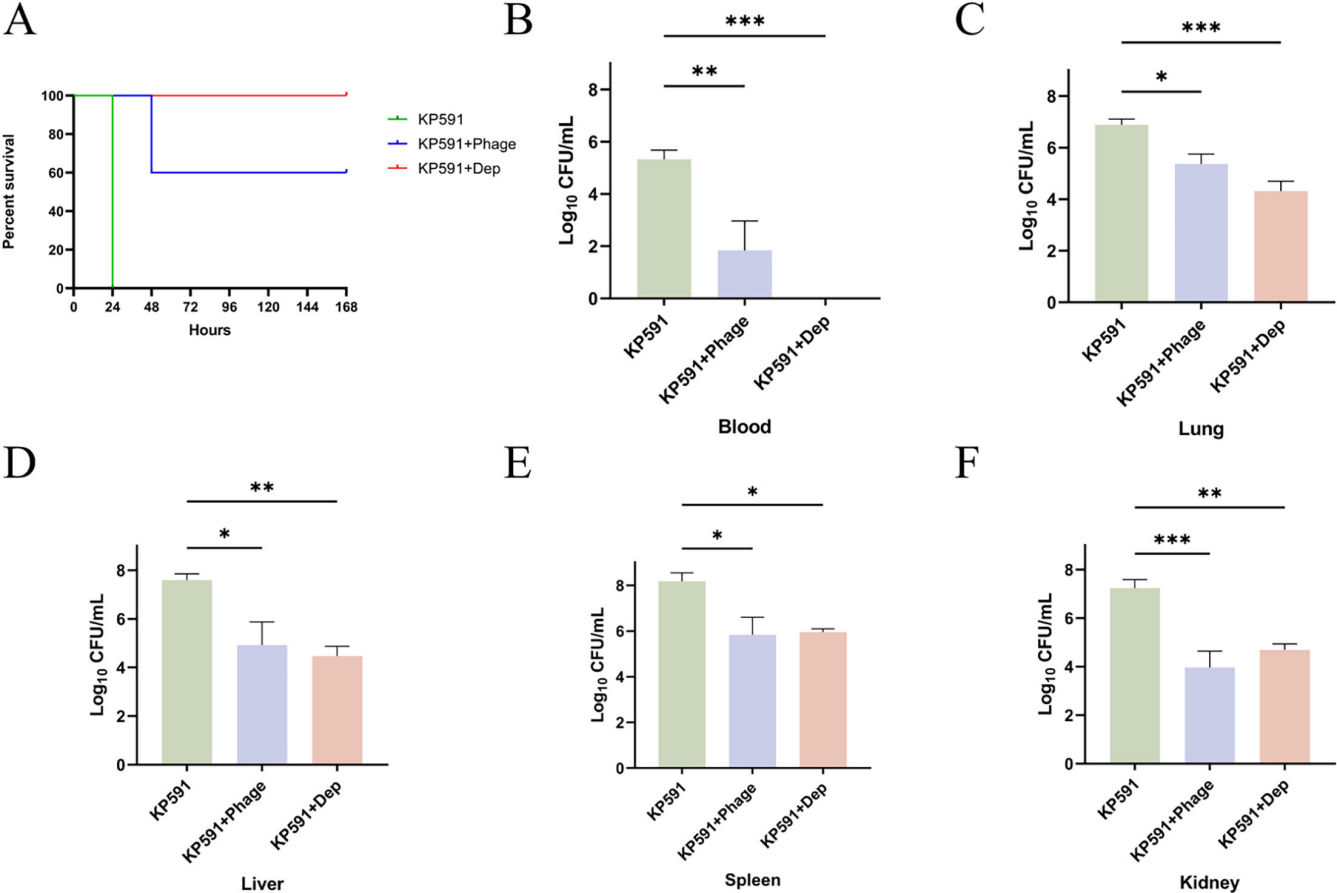
Assessment of the therapeutic efficacy of phage vB_Kp_Z57 and depolymerase DepZ57 in a mouse sepsis model. (**A**) Survival analysis of mice challenged with *K. pneumoniae* KP591. The survival rates were monitored over 168 h following treatment with PBS (Control, green line), phage (blue line), or DepZ57 (red line). (B–F) Quantification of bacterial burdens in the blood (**B**), lung (**C**), liver (**D**), spleen (**E**), and kidney (**F**). Bacterial loads are expressed as Log10 CFU/mL. Data are presented as mean ± standard deviation (SD). Asterisks indicate statistical significance compared to the untreated control group (*, *P* < 0.05, **, *P* < 0.01, ***, *P* < 0.001).

All untreated mice in the challenge control group died within 24 h post-infection. Over the 7-day observation period, DepZ57 treatment achieved a 100% survival rate, whereas phage treatment resulted in a 60% survival rate, with two deaths occurring within 48 h (Fig. 8A). By 12 h post-infection, systemic dissemination was evident in the challenge control group, with bacteria detected in the blood and all examined organs. Both DepZ57 and phage treatments significantly reduced bacterial loads in the blood, lungs, liver, spleen, and kidneys compared to the control. In the blood (control: 4.9 × 10^5^ CFU), DepZ57 achieved 100% reduction, outperforming the 96.4% reduction in the phage-treated group (Fig. 8B). In the lungs (control: 1.3 × 10⁷ CFU), phage and DepZ57 treatments reduced bacterial loads to 5.3 × 10^5^ CFU (95.8% reduction) and 7.1 × 10^4^ CFU (99.4% reduction), respectively (Fig. 8C). In the liver (control: 8.5 × 10^7^ CFU), phage and DepZ57 treatments reduced loads to 7.4 × 10^5^ CFU (99.1% reduction) and 1.7 × 10^4^ CFU (99.9% reduction), respectively (Fig. 8D). In the spleen (control: 3.1 × 10^8^ CFU), phage and DepZ57 reduced loads to 1.1 × 10^6^ CFU (99.6% reduction) and 1.9 × 10^6^ CFU (99.4% reduction), respectively (Fig. 8E). In the kidneys (control: 3.7 × 10^7^ CFU), bacterial loads decreased to 7.6 × 10^4^ CFU (99.8% reduction) with phage and 8.0 × 10^4^ CFU (99.8% reduction) with DepZ57 (Fig. 8F). Overall, DepZ57 demonstrated superior bacterial reduction in the blood, lungs, and liver, while showing comparable efficacy to phage treatment in the spleen and kidneys.

**Fig 9:**
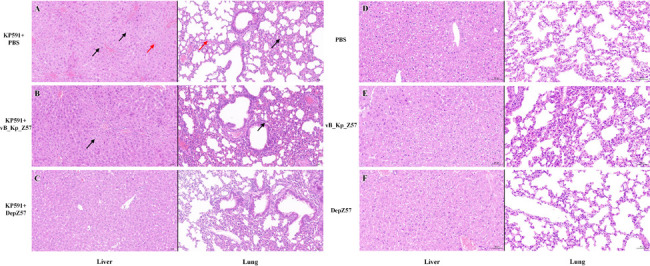
Histopathological assessment of therapeutic efficacy and safety *in vivo*. Representative hematoxylin and eosin (H&E)-stained sections of liver and lung tissues are shown for each group. (**A**) The infection control group was challenged with *K. pneumoniae* KP591 and treated with PBS. Lung: alveolar septal widening (black arrow) and inflammatory cell infiltration (red arrow). Liver: hepatocellular edema (black arrow) and venous congestion (red arrow). (**B**) The phage treatment group was challenged with KP591 and treated with vB_Kp_Z57. Lung: mild alveolar septal widening with scant inflammatory cell infiltration (black arrow). Liver: hepatocellular edema (black arrow). (**C**) The depolymerase treatment group was challenged with KP591 and treated with purified DepZ57, exhibiting significant alleviation of pathological lesions compared to the infection control. (**D**) The negative control group was inoculated with sterile PBS, serving as a healthy reference with normal histological structure. (**E**) The phage safety control group was inoculated with vB_Kp_Z57 only. (**F**) The depolymerase safety control group was inoculated with DepZ57 only. Scale bars represent 50 μm.

Livers from the challenge control group exhibited acute injury, characterized by extensive hepatocellular edema and severe venous congestion. Lungs in this group displayed typical features of bacterial pneumonia, including alveolar septal thickening, vascular congestion, and massive inflammatory cell infiltration (Fig. 9A). Phage treatment effectively mitigated tissue damage; livers maintained normal lobular architecture with localized periportal hepatocyte edema, while lungs remained structurally intact with mild alveolar septal thickening and minor inflammatory infiltration (Fig. 9B). Consistent with the phage treatment group, the liver lobular structure in the depolymerase treatment group was well preserved. Hepatocytes were arranged in orderly cords, and no obvious congestion or inflammatory infiltration was observed. The alveolar structure remained intact, with thin alveolar septa and clear alveolar spaces, and inflammatory cells were significantly reduced compared to the positive control group (Fig. 9C). Pathological analysis of the PBS-negative control group, the phage only group, and the depolymerase only group showed that the PBS control exhibited normal tissue morphology with no pathological changes (Fig. 9D). Crucially, the groups treated with phage only (Fig. 9E) and depolymerase only (Fig. 9F) showed histological features consistent with the PBS control. We observed no signs of inflammation, necrosis, or congestion in the liver or lung tissues of these groups.

## DISCUSSION

*Klebsiella pneumoniae* is an encapsulated gram-negative bacterium capable of causing diverse severe infections in both humans and animals. In clinical settings, it is a primary cause of bacteremia, pneumonia, meningitis, and pyogenic liver abscesses, whereas in veterinary contexts, it is a frequent causative agent of bovine mastitis and porcine pneumonia (38–41). Although animal-derived strains were generally considered to exhibit lower antibiotic resistance compared to human isolates, the extensive use of antibiotics has driven an increasing trend of resistance in these strains (42). Reports have identified MDR and hypervirulent strains in diverse animal hosts, including giraffes, elephants, rhinoceroses, and horses (43, 44). The global dissemination of these difficult-to-treat variants presents a severe challenge to clinical management, rendering therapeutic options critically scarce, particularly for strains resistant to carbapenems and polymyxins. In this study, we describe the isolation of a K57-specific lytic phage, vB_Kp_Z57, and the characterization of its encoded depolymerase, DepZ57. The findings presented herein suggest that DepZ57 represents a promising novel therapeutic candidate for the treatment of K57 *K. pneumoniae* infections.

In this study, the therapeutic efficacy of the phage and its encoded depolymerase was systematically compared in *in vitro* and *in vivo*. In a murine lethal sepsis model, DepZ57 provided 100% protection, whereas vB_Kp_Z57 achieved a survival rate of only 60%. This difference is attributable to their distinct mechanisms of interaction with both the pathogen and the host. Phage therapy relies on rapid bacterial lysis, which in severe systemic infections causes a sudden and massive release of endotoxins (LPS) (45). This abrupt surge may trigger a fatal cytokine storm and overwhelm an already compromised immune system (46, 47). Conversely, depolymerases lack direct bactericidal activity and work through a non-lytic mechanism, safely avoiding this endotoxin spike. Beyond mitigating endotoxin release, depolymerases synergize with innate immunity by reducing bacterial virulence. The capsular polysaccharide is a major virulence factor for *K. pneumoniae*, and degrading it attenuates the pathogen (48). This enzymatic decapsulation simultaneously exposes the bacterial surface structures to the host’s innate immune defenses, facilitating a steady, immune-mediated clearance through complement activation and macrophage phagocytosis (49). While whole phages also possess structural depolymerases, these function primarily as receptor-binding proteins for adsorption; their action is confined to the local bacterial microenvironment and may be insufficient to induce the widespread decapsulation achieved by the high-dose purified enzyme (50). Furthermore, the evolutionary dynamics of these treatments differ significantly. Actively replicating phages exert intense survival pressure on bacteria, driving the rapid appearance of resistant mutants within hours and adding an additional burden to the heavily infected host (51). By contrast, while depolymerase therapy could theoretically induce resistance mutations, its action is restricted to the capsule. Any capsule-altered mutants that emerge are typically attenuated in virulence and remain susceptible to immune clearance, thereby reducing the risk of resistance compromising therapeutic efficacy. These hypotheses were further corroborated by *in vitro* findings, which demonstrated that depolymerase-mediated CPS degradation sensitized the bacteria to phagocytosis and serum killing, indicating the capacity of the enzyme to recruit the host innate immune system. Although phage pre-treatment was also observed to induce serum sensitivity via CPS degradation, bacterial elimination was primarily driven by direct lytic activity. Notably, the rate of bacterial clearance by phages significantly exceeded that of the depolymerase-mediated immune clearance, a factor that could accelerate LPS release and potentially exacerbate systemic tissue damage.

Depolymerases typically consist of a conserved N-terminal domain and a variable C-terminal domain (52). Bioinformatic analysis revealed that DepZ57 possesses the modular architecture characteristic of tail fiber proteins. We hypothesize that the conserved N-terminal domain is responsible for anchoring the protein to the phage baseplate, while the variable C-terminal domain contains the catalytic center responsible for substrate recognition. This structural organization dictates the strict serotype specificity observed in our results. Previous studies involving domain swapping and gene deletion have confirmed that the C-terminal domain is the primary determinant of host specificity (53). The sequence conservation observed between the C-terminus of DepZ57 and other reported K57-targeting phage depolymerases further corroborates this conclusion. These findings suggest that the C-terminus of DepZ57 serves as the functional enzymatic domain. Notably, our strain collection encompassed K57 isolates from diverse hosts, including humans, swine, and bovines. DepZ57 effectively depolymerized the capsules of all these isolates regardless of their source. This capability highlights its broad utility, suggesting potential applications not only in human medicine but also in controlling zoonotic transmission. The development of a comprehensive library of depolymerases targeting various serotypes could significantly alleviate the pressure of multidrug-resistant bacteria on public health resources.

Enzymatic stability is a critical limiting factor in the translation of depolymerases into clinical drugs (54). In this study, DepZ57 exhibited remarkable physicochemical stability. DepZ57 retains activity at pH 2.0 and withstands temperatures up to 70°C. This tolerance to extreme conditions suggests significant advantages regarding industrial preparation and storage, and implies potential adaptability to the gastric environment for oral administration.

Regarding safety, the administration of high doses of either phage or DepZ57 alone did not induce any pathological changes or mortality in mice, confirming a favorable biosafety profile. However, this study has several limitations. First, we primarily evaluated efficacy via intraperitoneal administration; future studies should investigate alternative routes such as intravenous injection or aerosol inhalation. Additionally, while DepZ57 performed exceptionally well in a murine model, its clearance efficacy in other animal models requires verification. Given that DepZ57 assists immune clearance rather than directly killing bacteria, subsequent research should explore combining DepZ57 with antibiotics to investigate potential synergistic strategies, aiming to achieve optimal therapeutic efficacy even in immunocompromised patients.

In conclusion, this study identified a novel depolymerase, DepZ57, specific to the K57 serotype. With its superior protective efficacy *in vivo*, excellent stability across broad pH and temperature ranges, and favorable biosafety, DepZ57 stands as a potent candidate for controlling hypervirulent K57 *K. pneumoniae* infections, offering a new alternative for antimicrobial therapy in the post-antibiotic era.

## Data Availability

The complete genome sequence of phage vB_Kp_Z57 has been deposited in GenBank under the accession number PP758980. The gene encoding the tail tubular protein Gp12 spans the linearization point of the circular genome. In accordance with GenBank submission guidelines for linear representations of circular genomes, this gene is annotated as two discontinuous miscfeature regions (at the 3'- and 5'-ends) in the database record, but is described as a complete coding sequence in this article.
